# BIOMECHANICAL EVALUATION OF FIXATION METHODS IN PATELLAR FRACTURES (AO 34-C3): AN IN VITRO ANALYSIS

**DOI:** 10.1590/1413-785220263401e291017

**Published:** 2026-02-13

**Authors:** Marcelo de Almeida Ferrer, Silvio Leite de Macedo, Hebe Soledad Simões Gomes de Moura, Leonardo Rigobello Battaglion, Anderson Freitas, Bruno do Nascimento Ohashi

**Affiliations:** 1 Clinica Ortosul, Brasilia, DF, Brazil.; 2 Hospital Regional do Paranoa, Servico de Residencia Medica em Ortopedia e Traumatologia, Brasilia, DF, Brazil.; 3 Hospital Regional do Gama, Servico de Residencia Medica em Ortopedia e Traumatologia, Brasilia, DF, Brazil.; 4 Universidade de Sao Paulo, Faculdade de Medicina de Ribeirao Preto (USP), Ribeirao Preto, SP, Brazil.

**Keywords:** Finite Element Analysis, Fracturs Fixation, Patella, Bone Plates, Análise de Elementos Finitos, Fixação de Fratura, Patela, Placas Ósseas

## Abstract

**Objective:**

To describe the biomechanical outcomes of five different fixation models, namely: tension band wiring (TB), anterior star-shaped locking plate (PS), lateral orthogonal locking plates (LP), and two additional models using BT and LP combined with a circumferential cerclage wire around the patella, referred to as BTO and PSO, respectively, in a comminuted patellar fracture (AO34C3).

**Methods:**

This study analyzed, using the finite element method (FEM), the following variables: total displacement, fragment displacement, maximum and minimum principal stresses, and total and localized von Mises stresses, under loading conditions of 1,500 N (R1) and 3,000 N (R2).

**Results:**

Results were presented as absolute values and percentiles, as well as through observational analysis of individual stress distributions. From a biomechanical perspective, critical results in both absolute and percentile values were observed for most variables in the TB group compared with the PS and LP groups. While maximum and minimum principal stresses were similar among groups, differences in intensity and location were found for total and localized von Mises stresses. In addition, the inferior pole of the patella exhibited critical stress conditions across all groups.

**Conclusions:**

Tension band wiring demonstrated inferior outcomes compared with locking plates. Biomechanical benefits were observed with the use of two orthogonal locking plates compared with an anterior locking plate; however, both constructs still showed deficiencies in stabilizing the distal pole of the patella. ***Level of Evidence II; Prospective Study.***

## RESUMO

**Objetivo:**

Descrever os resultados biomecânicos de cinco diferentes modelos de fixação: banda de tensão (BT), placa bloqueada anterior em forma de estrela (PE), placas bloqueadas ortogonais laterais (PL) e dois modelos adicionais combinando BT e PE com fio de cerclagem circunferencial ao redor da patela, denominados BTO e PEO, respectivamente, em uma fratura cominutiva da patela (AO 34-C3).

**Métodos:**

Este estudo analisou, por meio do método dos elementos finitos (MEF), as seguintes variáveis: deslocamento total, deslocamento dos fragmentos, tensões principais máxima e mínima e tensões de von Mises total e localizada, sob condições de carregamento de 1.500 N (R1) e 3.000 N (R2).

**Resultados:**

Os resultados foram apresentados em valores absolutos e percentis, bem como por meio de análise observacional das distribuições individuais de tensão. Do ponto de vista biomecânico, observaram-se resultados críticos, em valores absolutos e percentis, para a maioria das variáveis no grupo BT em comparação aos grupos PE e PL. As tensões principais máxima e mínima foram semelhantes entre os grupos; entretanto, observaram-se diferenças na intensidade e na localização das tensões de von Mises total e localizada. Ademais, o polo inferior da patela apresentou condições críticas de tensão em todos os grupos.

**Conclusões:**

A banda de tensão apresentou desempenho biomecânico inferior quando comparada às placas bloqueadas. Benefícios biomecânicos foram observados com o uso de duas placas bloqueadas ortogonais em relação à placa bloqueada anterior; contudo, ambas as construções apresentaram limitações na estabilização do polo distal da patela. ***Nível de Evidência: II; Estudo Prospectivo*.**

## INTRODUCTION

The patella fracture represents 1% of the skeletal fractures, and can cause significant functional disabilities due to its relationship with the knee extension mechanism.^1–4^ The patella supports forces of 3.3 to 7 times the body weight,^5,6^ affecting the patellofemoral joint and resulting in comminuted fractures.^7,8^

It is a challenge for the surgeon to treat the biological damage and restore joint congruence, as well as maintain mechanical stability with stable synthesis.^9^ The forces in the patella affect the synthesis, which must withstand various biomechanical conditions of the knee (flexion, patellar tilt, tibial and femoral rotation).^10^

Some methods, such as the tension band, have complications (22-30%) due to release, wire migration and local irritation, requiring reinterventions in up to 65% of cases.^7,8,11–14^ The previous plates show more promising results, since in the comminuted fractures of the patella allow the individualized fixation of fragments with angular stability.^15,16^

Thus, the biomechanical benefits inherent in the use of plates, when compared to the use of Kirchner wires and voltage bands still need experimental studies making it possible to evaluate the most beneficial hypothesis from a biomechanical point of view.

The authors describe a biomechanical test, using the finite elements method, on a five-part patellar fracture (AO 34C3), fixed with five synthesis models, to evaluate total displacement, fragment displacement and tension distribution in the patella and in the syntheses, using Von Mises evaluation and presenting results in absolute values, percentiles and observational analysis of voltage distribution.

## METHODS

Tomographic images were obtained of a left patella with 45 mm wide, 43 mm long and 20 mm thick, extracted from the synthetic model 1145-70 (large size) of the brand SawboneTM, composed of cortical and polyurethane sponge. The images were archived in the DICOM protocol using an Emotion tomograph (16 channels, Siemens, Germany) with a resolution of 512×512 and a cut-off distance of 1.0 mm. The file was imported into the InVesalius^TM^ program for 3D reconstruction and archived in STL format. The study did not use data from humans or any living beings in the research, only synthetic and virtual models of a left patella as described above, thus being dispensed from terms of free and informed consent or approval by ethics committee.

The virtual 3D models of each system (bone, tendon and muscle) were made in the Rhinoceros^TM^ 6 program (Robert McNeel & Associates, USA) and the finite element method (FEM) test was performed in the SimLab^TM^ (HyperWorks, USA) using the solver Optistruct.

**Figure 1:**
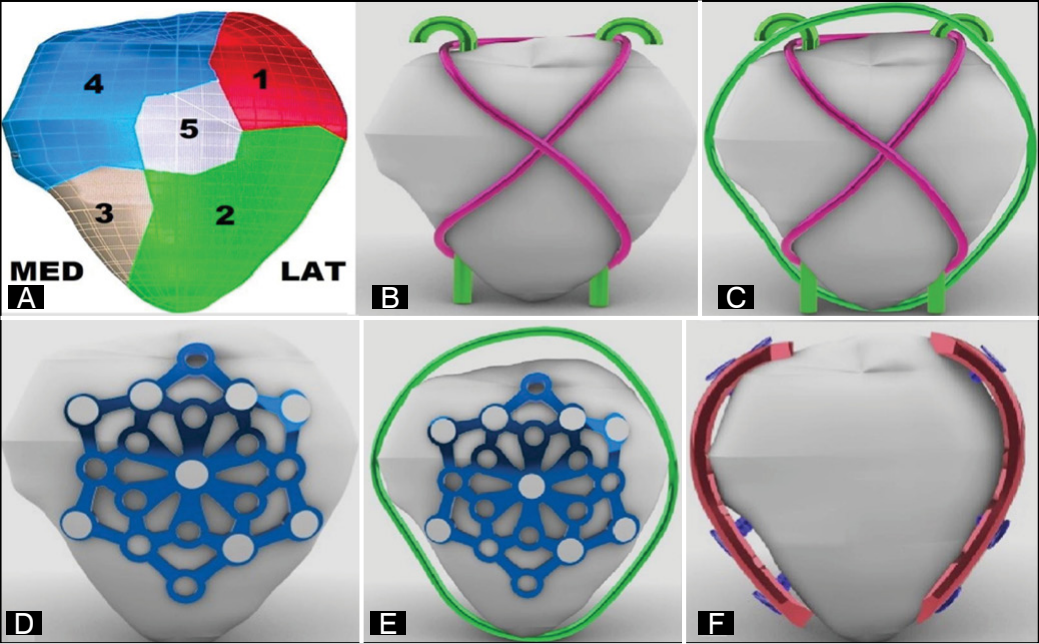
A) illustration demonstrating the 3D patella model and its respective fragments and faces. B) 3D model of the TB group. C) 3D model of the TBO group. D) 3D model of the PS group. E) 3D model of the PSO group. F) 3D model of the LP group.

Source: Author (Image generated in HyperView® (Altair) software).

Cuts were made in the patella on the axial and coronal axes, reproducing a fracture AO34C3, dividing the patella into five fragments (P): two in the upper pole, lateral (P1) medial (P4), two in the lower pole, lateral (P2) medial (P3) and a central (P5). The fragments had corresponding muscular and tendon inserts, being the higher to the quadricipital tendon (TQ) and the lower to the patellar tendon (TP), with representations of soft parts and a solution of discontinuity near the edge of fractures. (Figure 1A)

The synthesis models for fracture fixation were virtually drawn: Kirchner wire of 2.0 mm (K), circling wire of 1.25 mm (O), locked LCP plates of 2.7 mm with 4 holes (LP), star-shaped locked plate (PS) and screws of 2.7 mm. The dimensions corresponded to a physical model in 316L stainless steel from the brand DePuy Synthes (Switzerland).

The configured groups (n=1) were named according to the type of fixation: Group (TB) with tension band, using two parallel K wires and a circling wire in eight; (Figure 1B) group (PS) with a 2.7 mm star-shaped blocked plate on the front of the patella; (Fig. 1D) group (LP) with two 2.7 mm orthogonal blocked plates, one on the lateral side and one on the medial side of the patella (Figure 1F). Two other groups, TBO and PSO, added O wire to the TB and PS models, circumventing the patella and transferring TP and TQ. (Figure 1C,E) In the TB and TBO groups, the K wires were positioned parallelly in the axial plane for better bone contact area in the peripheral fragments (1, 2, 3, 4). The figure-of-eight tension band wire contacted the posterior surface of the K-wires at their ends, transmitting TQ and TP. In the LP, PS and PSO groups, the plates were positioned with at least two screws in each fragment (P). Angles and lengths of the screws respected the joint surface and sought the best area of bone contact. In the LP and LPO group, the screws reached the opposite side of the patella, respecting the cortical.

After composing the groups, all models were imported to Simlab^TM^ for trials. Each part of the models (cortical bone, sponge, tendon, muscle and steel) was identified and controlled for the mesh, keeping the size of the elements to avoid interference. A tetrahedral element was adopted, with a defined number of nodes and elements. For the simulations, the properties of the materials were defined: cortical bone (17,000 and 0.26), trabecular bone (1,700 and 0.26), patellar ligament and muscles (1,200 and 0.45) and steel alloy (200,000 and 0.29), referring to the elasticity module (Ma) and Poisson coefficient (v).

**Figure 2:**
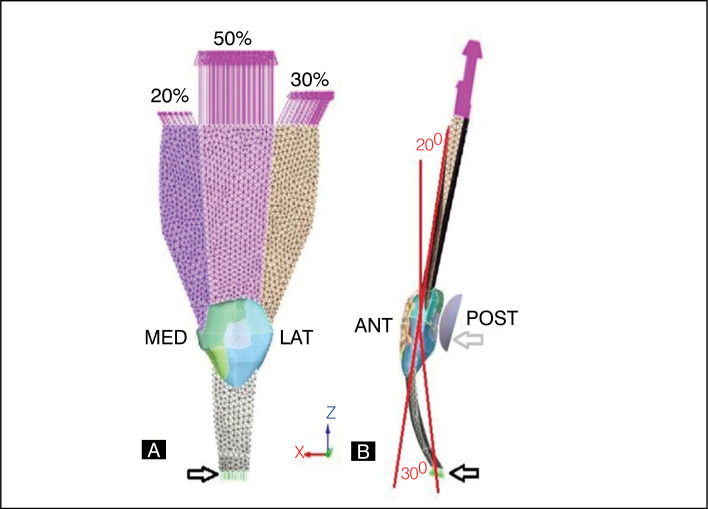
Conditions and contours of the tests. A) Front view (bottom to top) - Black Arrow - fixation point on the patellar tendon. Pink arrows - locations, axis, direction - fractions of charges applied to the portions of the quadriceps. B) Side view - (lower to top)- Black arrow - fixation point on the patellar tendon. red lines- Representation of the knee flexion angle in 20 degrees with long axis of the femur and 30 degrees with long axis of the tibia.- Grey arrow - femoral condyle.

Source: Author (Image generated in HyperView® (Altair) software).

The trials applied a traction force in the TQ, in the cranial direction in the coronal axis, distributing the total load into three fractions: 50% for the intermediate, 30% for the lateral and 20% for the medial, fixed at the distal end of the TP. Between the charging point and the insertion, there was a bending tilt of 20 degrees relative to the long axis of the femur and 30 degrees to the tibia, supported later by the femoral condyle, drawn according to the patellar joint surface to optimize joint contact in the predetermined angles. (Figure 2A,B) Two loads on the Z-axis were applied to all groups: 1,500 N (R1) and 3,000 N (R2) for physiological and supraphysiologic evaluation. On axes X and Y, there was no charge. Movement restriction regions were determined in the test body and femoral condyle in all directions of the X, Y and Z axes to avoid unwanted displacement and rotations. (Figure 2)

From these conditions, the values of total patella displacement (Desl.T) in mm and localized displacement (Desl.L) in mm were obtained, evaluated individually between the adjacent fragments (PI to P2, P4 and P5; P2 to P3 and P5; P3 to P4 and P5; P4 to P5). The main maximum voltage (Max.P) and the main minimum voltage (Min.P) were also measured, both in MPa, in addition to the total Von Mises voltage (VT) and localized (VL) in the syntheses. The results were presented in absolute values, percentiles and observational analysis of the distribution of tensions.

## RESULTS

When we observe Desl. T in R1, the values obtained were: 93.45; 80.47; 87.98; 80.12; 24.65 and in R2:156.14; 135.45; 146.63; 135.99; 43.19 (mm) for TB, TBO, PS, PSO and LP, respectively. The LP group had the lowest value and TB the highest values in both loads. TBO and PSO groups showed reduction relative to their individual pairs, and PS group had higher values compared to TBO group (Table 1). In relation to the percentile of LP and PS, 356% values were observed in R1 and 339% in R2 higher for PS compared to LP. In Desl. L, the fragments of the upper pole showed greater displacements compared to those of the lower pole (P1-P2 and P3-P4). The LP group presented the lowest absolute values. There was a reduction of this variable in the TBO and PSO groups relative to their individual pairs. The PS group had lower values, but close to those of the TBO group in R1, and lower values in R2. (Table 1) THE Max. P, which represents the traction force and its deformities, showed that the values in the LP group were superior to the PS and TB groups in R1 and R2. The TBO group had a lower value than the PSO, indicating lower distribution performance of the tensions in the TB and TBO groups. VT values were: 419.68; 359.97; 1805.53 (MPa) in R1 and 557.2; 720.19; 2171.06 (MPa) in R2, for LP, PS and TB, respectively. This indicates that the tension distribution in the syntheses was more effective in the LP group in R1 and PS in R2 (Table 1), with higher percentiles of LP versus PS at 85.77% in R1 and lower at 129.25% in R2.

**Table 1:** Variable values by group and load.

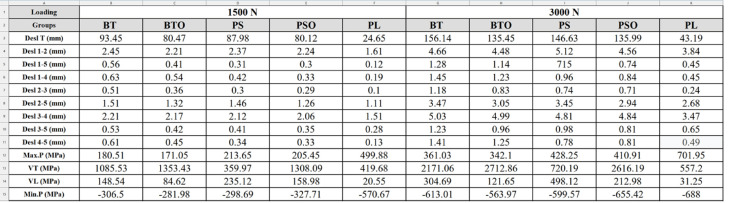

**Figure 3:**
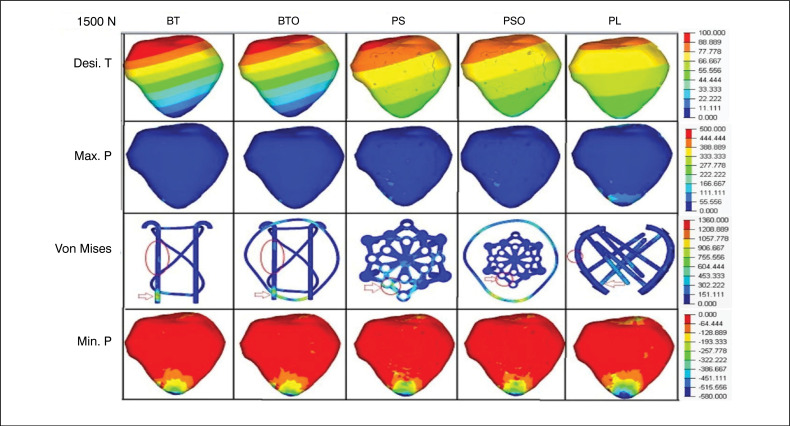
Loading 1500N- Images of the groups and variables with their respective grading scales. (red arrow) - Von Mises total. (red circle) - Von Mises located.

Source: Author (Image generated in HyperView® (Altair) software).

**Figure 4:**
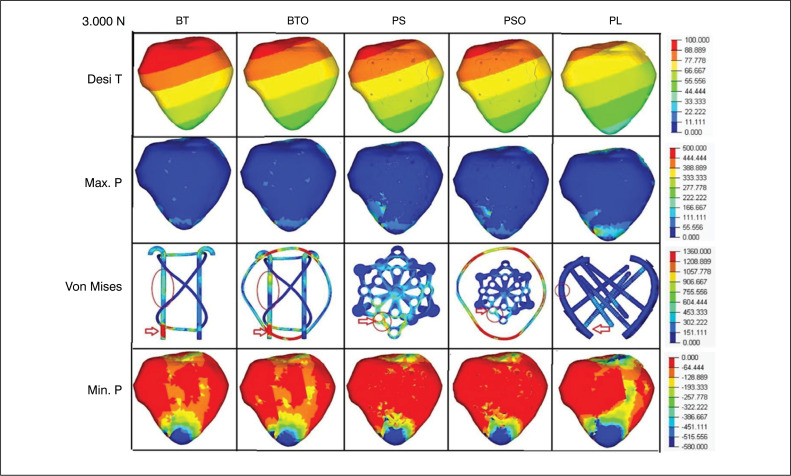
Loading 3000N- Images of the groups and variables with their respective grading scales. (red arrow) - Von Mises total. (red circle) - Von Mises located.

Source: Author (Image generated in HyperView® (Altair) software).

In the descriptive analysis of VT, especially in R2, the tension was located in the synthesis in all groups at the inferior-medial pole of the patella, P4. In the TB and TBO groups, the voltage was at the distal end of the medial K wire, in the PS and PSO groups, in the peripheral connection hole at the northwestern tip of the star, and in the LP group, at the base of the screws in contact with the medial plate. The voltage was distributed to the wire "O" in the TBO and PSO groups, reducing the absolute value without changing the location in R1 and R2. (Fig. 3 and 4)

VL values consolidate the results of the variables described, linked to the critical point of tension (break). In R1, the values were: 20.55; 235.12; 148.54 (MPa) and in R2:31.25; 498.12; 304.69 (MPa), for LP, PS and TB, respectively. This indicates effectiveness in the voltage distribution in the LP group, with lower values compared to the PS group, similar in R1 and R2 (Table 1), with percentiles of LP versus PS of 1,144% in R1 and 1,593% in R2.

In the descriptive analysis of VL in R2, there was similarity in the location of this variable with the VT in the PS and PSO group, concentrated in the peripheral connection hole of the northwestern tip of the star, P4. In the TB, TBO and LP groups, there was migration to the median third of the medial K thread in TB and TBO, and to the median third of the medial plate in LP. The distribution of voltage to the wire "O" in the TBO and PSO groups resulted in a reduction compared to the pair without "O". (Fig. 3 and 4)

For Min.P, represented in negative values to indicate direction and direction opposite to Max.P, we observed that the values in the LP group were superior to the PS and TB groups in R1 and R2. The TBO group had a lower value than the PSO, indicating lower distribution performance of the tensions in the TB and TBO groups. Min.T values were:570,67;-298,69;-306,5(MPa) in R1 and −688-599,57;-613.01 (MPa) in R2, for LP, PS and TB, respectively. Compression voltage distribution was most effective in the LP group, similar in R1 and R2. The distribution to the wire "O" was also observed in the TBO and PSO groups, reducing the voltage compared to the pair without "O". (Table 1) (Figures 3 and 4)

## DISCUSSION

This study evaluated five types of biomechanical fixation in the patella fracture 34C3 in five parts using FEM. Obtaining as main results less deviation from the fractured fragments, better stability, with the uses of orthogonal or previous plates when compared with the tension band, these results are similar to those obtained by Kfuri et al.^15^

The method allowed to compare quantitative and observational variables, focusing on the distribution of tensions, this visual approach helps to understand the behavior of the variables in the fixation groups of these complex fractures, bringing to light observational possibilities only possible with the use of FEM.

Biomechanical studies of comminuted patella fractures are rare in the literature, ^15–17^ possibly due to the reproduction difficulties of these tests, these difficulties minimized by the use of FEM. This method can reproduce complex conditions and is widely validated in traumatology.^18,19^

The biomechanics and anatomy of the knee are complex, involving multiple intrinsic variables, musculature, ligaments and adjacent joints. Even with the FEM, the reproduction of these conditions is a search for accurate information for research, considering various descriptions of conditions, contours, positioning, values and axes of loads, influential anatomical structures, among others. In addition to the FEM, counter tests are also important, although they may present vulnerabilities and biases.

The authors applied anatomical and biomechanical details, considering the patella and the patellofemoral joint and their possible deformations. They included the fractional application of the load on the femoral quadriceps and its axes described by Mesfar et.al.^20^ total load between three times the body weight of an adult from 50 to 100 kg, described by Zderic et.al. 2017,^6^ angles of the load and the degree of flexion of the knee with the FEM, described by Ling et.al.^21^ These criteria were selected by associating significant load to the patella and patellofemoral joint at their moment of highest tension.^22,23^ It is possible to present some comparative observations to them, which are: the presence of excessive Desl.T in the group TB in relation to the other groups, linked mainly to the fragments of the upper pole(1-4) in relation to the lower(2-3) and the medial fragments (3-4) in relation to the laterals(1-2), similar to the one described by Kfuri et al.^15,12,24^ with smaller displacements to the groups with blocked plates.

The lower pole of the patella was close to some form of fixation in the PAO and TBO groups, showing tension in the wire "O" in the transfixation area, resulting in tensional changes and small displacement reductions. Stoffel et al.^17,24^

This study presents limitations, as it did not consider the chondral component of the patellofemoral surface, the surface of the femoral condyle, the mechanical properties of the synovial liquid, the actions of ligaments and meniscal structure. It also considered only one angle of bending of the knee, restricting the trial on the three axes and preventing the rotational mobility of the patella. It is also worth mentioning as a limitation the absence of other fixation methods used in the treatment of this type of fracture (canular screw + tension band).

As strengths, attention is highlighted to the details of conditions and contours, considering the most important factors for this type of study, and the observational similarities with research using fresh cadaveric models. Future studies should contemplate new designs of previous blocked plates, which can optimize stability in areas of demonstrated fragility.

## CONCLUSION

In the fractures 34C3 of the patella evaluated by the FEM, the tension band has lower results when compared to the blocked plates, and biomechanic benefits were perceived in fixing this fracture model when we used two orthogonal plates blocked on the sides (medial and lateral) of the patella and when used the previous blocked plate, but both still with deficiencies in the stabilization of the distal pole.

## Data Availability

The data will be made available when requested.
